# A case of a complete atrioventricular canal defect in a ferret

**DOI:** 10.1186/s12917-020-02736-2

**Published:** 2021-01-22

**Authors:** Carlos F Agudelo, Vladimír Jekl, Karel Hauptman, Michal Crha, Meric Kocaturk

**Affiliations:** 1 Small Animal Clinic, Faculty of Veterinary Medicine, University of Veterinary and Pharmaceutical Sciences Brno, Palackého tř. 1946/1, 612 42, Brno, Czech Republic; 2 Department of Pharmacology and Pharmacy, Faculty of Veterinary Medicine, University of Veterinary and Pharmaceutical Sciences Brno, Brno, Czech Republic; 3 Jekl & Hauptman Veterinary Clinic, Brno, Czech Republic; 4 Department of Internal Medicine, Uludag University Faculty of Veterinary Medicine, Bursa, Turkey

**Keywords:** Atrioventricular canal, Pet ferret, Endocardial cushion defect, Heart failure, Echocardiography

## Abstract

**Background:**

Atrioventricular canal defect is a rare congenital disorder of the heart and describes the presence of an atrial septal defect, a variable presentation of ventricular septal alterations including ventricular septal defect malformations in the mitral and tricuspid valves. The defect has been described in human beings, dogs, cats, pigs, and horses.

**Case presentation:**

This paper describes the case of a complete atrioventricular canal defect in a four-year-old intact male pet ferret (*Mustela putorius furo*), which was presented due to posterior weakness, ataxia, and decreased appetite. A loud systolic murmur, dyspnea, and hind limb paraparesis were detected during the clinical examination. Thoracic radiographs showed generalized cardiomegaly and lung edema. ECG showed sinus rhythm with prolonged P waves and QRS complexes. Echocardiography showed a large atrial septal defect, atrioventricular dysplasia, and a ventricular septal defect. Palliative treatment with oxygen, furosemide, spironolactone, enalapril, diltiazem, and supportive care was chosen as the therapy of choice. The ferret recovered gradually during hospitalization. A follow-up examination at three and six months showed stabilization of cardiac function.

**Conclusions:**

To the authors knowledge, this is the first time an atrioventricular canal defect has been described in a pet ferret.

## Background

An atrioventricular canal defect (AVCD) is a rare congenital disorder of the heart characterized by an ostium primum atrial septal defect (ASD), alterations of the ventricular septum, like ventricular septal defect (VSD), and defects in the atrioventricular valves (AV) [1]. It occurs due to the failure of the development of the endocardial cushions during embryogenesis and the persistence of a primitive single AV canal [2].

An AVCD may be partial (incomplete), transient or intermediate, transitional or complete. The difference between them also reflects the severity of the defect [1–4]. The complete variety results from concurrent large ASD and VSD (i.e. common canal) as well as AV dysplasia. Typically, there is a large (uninterrupted) communication between all four heart chambers. A transient AVCD has common AV that are often inserted into the IVS that partially close the gap between the chambers. The VSD is often small with a moderate restriction of blood flow. An incomplete AVCD occurs when the flaps of the AV valves are connected to an interatrial or IVS without an identifiable VSD [1, 3].

In veterinary medicine, AVCD has been described in several species: eight pigs [5], 31 cats [1, 6–9], nine dogs [2–4, 10–14], and four horses [15–18]. Factors like oxygen deficiency, viral infection during early pregnancy, nutritional, genetic, and teratogenic factors have been suggested as possible factors triggering the development of this condition and possibly other congenital heart diseases [14, 18]. To the author’s knowledge, this is the first case of an incomplete AV canal in a ferret that has survived six months after the diagnosis with conservative therapy.

## Case presentation

**Fig. 1:**
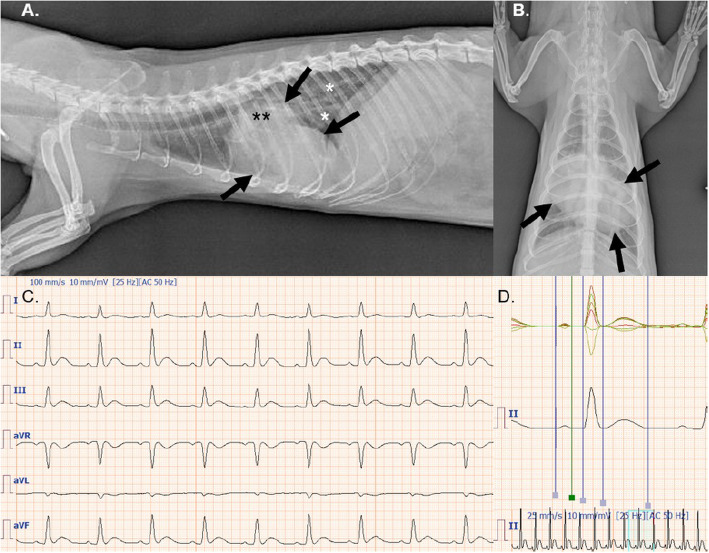
**a** Left laterolateral projection shows generalized cardiomegaly (vertebral heart score = 8) namely both ventricles and the left atrium (black arrows) with dorsal displacement of the trachea. There is also venous congestion (black asterisks) and bronchial lung pattern presence on the caudal lung lobes (white asterisks). **b** The dorsoventral view also demonstrates marked biventricular and left atrial enlargement. **c** Sinus rhythm (regular RR intervals) at a rate of 200–210 bpm. 100 mm / s and 1 cm = 10 mV. **d** Digital simultaneous superimposition of limb leads enhancing the measurement of diferent waves and intervals. There is a prolongation of the P wave suggesting left atrial (0.04 s; normal 0.01–0.03 s) and left ventricular enlargement (0.055 s; normal 0.02–0.05 s) [19]

A four-year-old intact male pet ferret (*Mustela putorius furo*) was presented to the Avian and Exotic Animal Clinic at the University of Veterinary and Pharmaceutical Sciences Brno, Czech Republic with a seven-day history of posterior weakness, ataxia, and decreased appetite. Clinical examination revealed lethargy, hind limb paraparesis, prolonged capillary refill time (three seconds), labored respiration, and dental tartar. A marked cardiac systolic murmur (grade 5/6) was found during thoracic auscultation. Jugular vein distention was not evident. Occasional splitting of the 1st heart sound was also noted, and abdominal palpation revealed splenomegaly. Complete cell blood count, plasma biochemistry, and cardiological examination that included thoracic radiography, electrocardiography (ECG), and echocardiography were performed. Laboratory analysis showed mild hyperphosphatemia and slightly increased blood urea concentrations. Radiographs showed an enlarged cardiac silhouette (vertebral heart score 8; reference range 3.75 to 4.07) [19], bronchial pulmonary pattern, and venous congestion (Fig. 1a, b). An ECG taken in right lateral position recorded sinus rhythm with possible left atrial and left ventricular enlargement (Fig. 1c, d).

**Fig. 2:**
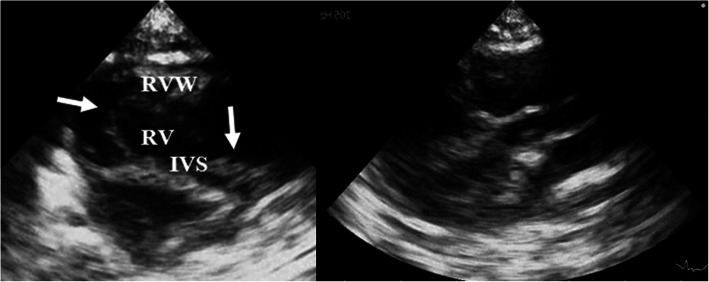
Left: Right parasternal short axis view displaying right ventricular hypertrophy and IVS at the level of papillary muscles. Flattening of the interventricular septum is present. There is present tricuspid valve deformation showing abnormal inserted portions of the valve leaflets (white arrows). Right: Mildly anteriorly displaced aortic valve with elongated LVOT (goose neck). Key: IVS: interventricular septum; RV: right ventricle; RVW: right ventricular wall

Echocardiographic examination was performed from all standard views, using an Aloka Prosound alpha 7 ultrasound machine (Hitachi Aloka Medical, Tokyo, Japan) equipped with a phased array 2–9 MHz cardio probe. Hypertrophy of the left ventricular posterior wall (diastole 6.1 mm), hypertrophy of papillary muscles and eccentric right ventricular hypertrophy with marked myocardial hypertrophy of the right ventricular wall (Fig. 2) were revealed from a right parasternal short axis view. Also, abnormal tricuspid leaflets were observed.

**Fig. 3:**
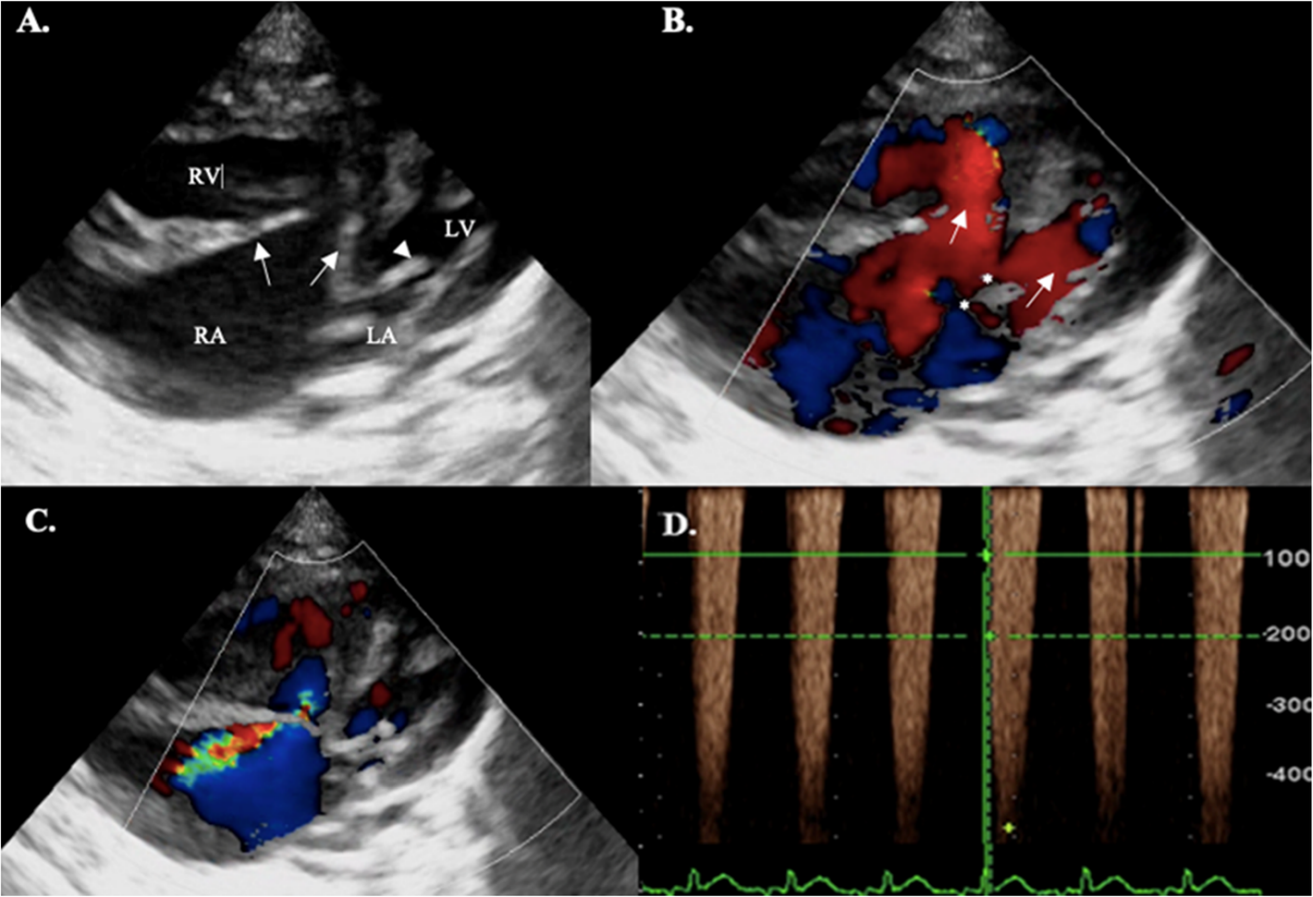
Simultaneous B-mode, CFM and CW imaging in a modified left apical 5-chamber view. **a** Marked right atrial enlargement can be noticed with a markedly thickened tricuspidal leaflets (white arrows) and absence of the atrial septum. Anterior mitral leaflet (white arrowhead). **b** AV inflow shows common filling of both ventricles (white arrows) and defects position (asteriscs). **c** CFM exam shows a large tricuspid regurgitation that reach portions of the left atrium. **d** CW Doppler. The cursor is located across the tricuspid regurgitation at the beginning of the systole and shows a velocity of 4.39 m/s (82 mmHg). Key: LA: left atrium; RA: right atrium, RV: right ventricle

Right parasternal long axis and modified left apical views revealed a single very large atrium due to a large ostium primum ASD, a left-to-right VSD, a common AV that inserts in the crest of the IVS, and abnormal morphology of the tricuspid valve that displayed one leaflet abnormally prominent arising from the free wall of the right ventricle (Fig. 3a). The mitral valve seemed normal. Color flow mapping (CFM) demonstrated tricuspid and mitral insufficiency, and blood flowing between the left and right part of the single atrium (Fig. 3b, c). The Qp / Qs determined by measuring Doppler-derived aortic and pulmonary integral systolic flow rates was 1.6, indicating increased right cardiac output. Continuous-wave Doppler analysis of the tricuspid jet revealed severe pulmonary arterial hypertension with a peak velocity of 4.39 m/s (Fig. 3d). A diagnosis of a complete AVCD Rastelli type A defect was determined based on the echocardiographic findings, which confirmed the presence of a large ostium primum ASD, VSD and dysplastic AV valves.

Initial therapy consisted of oxygen therapy and intravenous isotonic fluids. After final diagnosis, medical therapy was started with furosemide (4 mg/kg IV, QID), spironolactone (12.5 mg/kg PO, BID), enalapril (0.5 mg/kg PO, SID) and diltiazem (7.5 mg/kg PO, BID). Two days after, the patient was discharged from hospitalization with the above-mentioned therapy, except the furosemide that was given orally (4 mg/kg q24h). At a two-week follow-up, the ferret’s condition had improved and was able to walk and eat normally. Administration of furosemide and diltiazem was discontinued after two weeks and five weeks, respectively. The last follow-up examination at 6 months showed stabilization of cardiac function without significant changes on ECG and echocardiographic examinations.

## Discussion and conclusions

Complete AVCD is a very rare finding in all mammal species but, according to literature, may be found relatively common in cats and pigs. One paper reports about 8 cases in porcine that can be assumed as a complete AVCD or at least having the intermediate type, where interatrial and interventricular defects were frequently accompanied by other defects like subaortic or pulmonic stenosis, vegetative endocarditis, or persistent left anterior vena cava [5]. Some cats were reported to have complete AVCD Rastelli type A and B [1]. Other 26 feline patients were diagnosed as incomplete AVCD in some instances (8/26) associated with other congenital heart abnormalities [1, 6–9]. Additionally, abnormalities of the tricuspid valve similar as to what observed in the presented case were observed in cats, where a large leaflet of the valve was more prominent [7]. Dogs were reported mainly with incomplete AVCD and, strikingly, in much lesser proportion than cats [2–4, 10–14]. In horses, only complete AVCD cases were described [15–18]. To date, there is no published information about the incidence of this defect in exotic companion mammals.

The development of clinical signs depends on the morphology of the heart defect, its size, and pressure gradient between the left and right side. Generally large defects result in marked right ventricular eccentric hypertrophy because the right ventricle is more compliant than the left ventricle in accommodating more blood volume if there is no interatrial septum [9]. This often may cause shunt reversal and poor prognosis. In this patient, there were several clinical signs of heart failure mainly due to the complete characteristics of AVCD. In general, incomplete AVCD does not cause clinical signs unless marked mitral valve regurgitation develops [1]. If heart failure is present, arrhythmias, respiratory difficulty due to pulmonary edema or pleural and abdominal effusion may be present. Feline patients with severe AV canal defects generally die due to secondary left heart failure [7]. This patient was presented with severe left-sided and right-sided heart failure that improved after medical therapy. The authors have observed hind limb paresis as a common sign of heart disease in ferrets, which is unrelated to arterial thromboembolism but with limb weakness [19]. This ferret was free from clinical symptoms for at least six months after diagnosis. According to our clinical experience, patients with left-to-right shunting clinically improve or, at least, do not worsen after beta-blocker therapy when titrated gradually.

A diagnosis of AVCD is performed based on cardiovascular examination using imaging methods like radiography, ECG, and echocardiography. Generally, radiography reveals cardiomegaly, especially regarding the right atrium, both ventricles, and a pronounced pulmonary artery [4, 14]. The ECG commonly shows QRS changes compatible with conduction abnormalities, especially bundle branch blocks [3]. However, in the presented case, observed changes were related to the left atrial enlargement (prolonged P waves) probably due to volume overload in the single atrium and increased pressure in pulmonary veins associated with clinical signs of left-sided heart failure. Two-dimensional echocardiography can reveal the presence and size of ASD and VSD and allows the assessment of the AV morphology and the papillary muscles architecture [1]. Although this ferret had several malformations, the left ventricle function was sufficient to provide normal blood pressure and peripheral perfusion. The presence of simultaneous ASD, VSD, tricuspid valve changes and AV insufficiency helped to differentiate complete AVCD from other types of AVCD, common atrium and incomplete ASD associated with mitral and tricuspid dysplasia [4]. Severe systolic pulmonary hypertension was determined by right ventricular hypertrophy and high-velocity tricuspid regurgitation with a peak velocity of 4.39 m/s (82 mm Hg, assuming a right atrial systolic pressure of five mm Hg) that certainly contributed to the complexity of the clinical signs.

Medical management is aimed at slow cardiac remodeling, preserving ventricular function and controlling signs of congestive heart failure with ACE inhibitors, beta-blockers, spironolactone, diuretics, exercise restriction, oxygen, and supportive therapy. In our case, early diuretic, inotropic and vasodilatory therapy significantly reduced left and right ventricular overload and postponed disease progression. Open cardiac surgery for AVCD using extracorporeal circulation has been described in several case reports in dogs and cats [11–13]. Due to the relatively small body size of the patient, and also the high price of the procedure, the owner declined this alternative.

In conclusion, a complete AVCD characterized by a large ostium primum ASD, VSD showing left-to-right shunting and AV dysplasia in a ferret showed clinical signs of heart failure according to history and clinical signs. The final diagnosis was achieved by echocardiography that demonstrated the complex lesions. The ferret survived at least 6 months with only medical treatment suggesting that this approach could be considered in other severe heart congenital heart disease in other species.

## Data Availability

Data is available at the Small Animal Clinic (internal medicine and diagnostic imaging departments) at the University of Veterinary and Pharmaceutical Sciences Brno.

## Abbreviations

AVCD: Atrioventricular canal defect
ASD: Atrial septal defect
AV: Atrioventricular valves
IVS: Ventricular septum
VSD: Ventricular septal defect

## References

[1] Schrope DP (2013). Atrioventricular septal defects: natural history, echocardiographic, electrocardiographic, and radiographic findings in 26 cats. J Vet Cardiol.

[2] Saponaro V, Staffieri F, Franchini D, Crovace A (2010). Complete atrioventricular canal in a dog. J Vet Cardiol.

[3] Lee SG, Nam SJ, Moon HS, Hyun CB (2008). Partial atrioventricular canal defect in a maltese dog. J Vet Clin.

[4] Amberger CN, Boujon CE, Amberger C, Boujon C (2002). Atrioventricular canal defect (incomplete form of endocardial cushion defect) and ostium secundum type interatrial septal defect in a dog. J Vet Cardiol.

[5] Hsu FS, Du SJ (1982). Congenital heart diseases in swine. Vet Pathol.

[6] Schulze T (2006). Incomplete atrioventricular canal defect in a British Blue Shorthair cat. UK Vet.

[7] Liu SK, Ettinger S (1968). Persistent common atrioventricular canal in two cats. J Am Vet Med Assoc.

[8] Nakao S, Tanaka R, Hamabe L, Suzuki S, Hsu HC, Fukushima R. N. Machida. Cor triatriatum sinister with incomplete atrioventricular septal defect in a cat. J Feline Med Surg. 2011;13:pp.- 463–6. 10.1016/j.jfms.2011.01.016 PMC10832718 21497529

[9] Block CL, Glassman MM (2019). Pulmonary artery banding in a kitten with a partial atrioventricular septal defect. J Vet Cardiol.

[10] Monnet E, Orton EC, Gaynor J, Boon J, Peterson D, Guadagnoli M. Diagnosis and surgical repair of partial atrioventricular septal defects in two dogs. J Am Vet Med Assoc. 1997;211:pp.- 569–72. 9290821

[11] Santamarina G, Espino L, Vila M, Suarez ML (2002). Partial atrioventricular canal defect in dog. J Small Anim Pract.

[12] Akiyama M, Tanaka R, Maruo K, Yamane Y (2005). Surgical Correction of partial atrioventricular septal defect with a ventricular septal defect in dog. J Am Anim Hosp Assoc.

[13] Nakayama T, Wakao Y, Uechi M, Muto M, Kageyama T, Tanaka K, Kawabata M, Takahashi M. A case report of surgical treatment of a dog with atrioventricular septal defect (incomplete form of endocardial cushion defect). J Ved Med Sci. 1994;56:pp.- 981–4. 10.1292/jvms.56.981 7865605

[14] Ohad DG, Baruch S, Perl S (2007). Incomplete atrioventricular canal complicated by cardiac tamponade and bidirectional shunting in an adult dog. J Am Anim Hosp Assoc.

[15] Kraus MS, Pariaut R, Alcaraz A, Gelzer AR, Malik N, Renaud-Farrell S, Charter ME, Fox PR, Moïse NS (2005). Complete atrioventricular canal defect in a foal: Clinical and pathological features. J Vet Cardiol.

[16] Kutasi O, Vörös K, Biksi I, Szenci O, Sötonyi P (2007). Common atrioventricular canal in a newborn foal: case report and review of the literature. Acta Vet Hung.

[17] Ecke P, Malik R, Kannegieter NJ (1991). Common atrioventricular canal in a foal. N Z Vet J.

[18] Spiro I (2002). Hematuria and a complex congenital heart defect in a newborn foal. Can Vet J.

[19] Wagner RA (2009). Ferret Cardiology. Vet Clin North Am Exot Anim Pract.

